# Illness perception, family resilience, and emotional distress among hospitalized older adults with multimorbidity in Xinjiang, China: a latent profile and mediation analysis

**DOI:** 10.3389/fpsyt.2026.1804083

**Published:** 2026-07-21

**Authors:** Weilong Wang, Runjia Zhang, Jiaqi Deng, Yongle Pei, Zhengwei Luo, Qian Yu, Xuexia Yang, Shufeng Ren, Qianqian Jia, Xiaoxuan He, Jingwen Cai, Bo Zhang, Wen Cai

**Affiliations:** 1 Department of Nursing, Xinjiang Medical University, Urumqi, Xinjiang, China; 2 Department of Nursing, The Fourth Affiliated Hospital of Xinjiang Medical University, Urumqi, Xinjiang, China; 3 Health Care Research Center for Xinjiang Regional population, Urumqi, China

**Keywords:** emotional distress, family resilience, illness perception, latent profile analysis, mediation analysis, multimorbidity

## Abstract

**Background:**

Older adults with multimorbidity frequently encounter significant psychological challenges, with negative illness perception being a prevalent issue. Such perceptions can intensify emotional distress. Family resilience—the ability of a family system to adapt positively to adversity—plays a crucial role in alleviating negative emotions, including anxiety and depression. This study aimed to investigate the heterogeneity of illness perception among older patients with multimorbidity and to assess the mediating effect of family resilience on the relationship between illness perception and emotional distress.

**Materials and Methods:**

Between October 2025 and January 2026, 420 multimorbid older patients were recruited in three tertiary hospitals of Xinjiang Province. The evaluation was done based on the Brief Illness Perception Questionnaire, the Chinese version of the Family Resilience Assessment Scale, and the Hospital Anxiety and Depression Scale. The two methods of analysis were the latent profile analysis used to determine the unique patterns of illness perception and the bootstrap-based mediation analysis to determine whether family resilience mediates.

**Results:**

Three distinct illness perception profiles were identified: low (32.6%), moderate (47.6%), and high (19.8%) illness perceptions. Using the low illness perception group as the reference, family resilience showed a significant partial mediating effect between moderate illness perception and anxiety (indirect effect = 0.510, 95% *CI*: 0.325–0.724), moderate illness perception and depression (indirect effect = 0.450, 95% *CI*: 0.283–0.631), high illness perception and anxiety (indirect effect = 1.263, 95% *CI*: 0.819–1.739), and high illness perception and depression (indirect effect = 1.114, 95% *CI*: 0.707–1.555).

**Discussion:**

The results demonstrated heterogeneity in the perception of illness among older multimorbid patients, as a substantial percentage of the sample had moderate-to-high levels. Family resilience played a major partial mediating role between illness perception and emotional distress. The findings suggest the significance of shifting the clinical emphasis to the family system and integrating family resilience improvement as one of the psychological support measures for chronic patients.

## 1 Introduction

In recent decades, the prevalence of multimorbidity, defined as the co-occurrence of two or more chronic medical conditions in an individual, has increased due to population aging and increased life expectancy globally (1). Multi-country studies have reported that the self-reported prevalence of multimorbidity increased from 12.7% in 2001 to 16.2% in 2011 (2). Moreover, driven by extended life expectancy and often limited public health resources, developing countries are experiencing a rapid rise in multimorbidity rates (3, 4). Indicatively, when a study about the China Health and Retirement Longitudinal Survey (CHARLS) database was conducted on 10,479 adults aged 60 years and above, multimorbidity prevalence among the Chinese elderly individuals was found to be 65.6% (5).

The emotional distress is common in older adults having numerous morbidities, which are mostly of a negative nature, including anxiety and depression (6, 7). According to a meta-analysis, the prevalence of depressive symptoms in older adults with multimorbidity was 32.4% worldwide, and the prevalence of anxiety symptoms was 27.8% (8). Notably, the prevalence of depressive symptoms among older Chinese adults with multimorbidity was 35.6%, which is higher than the global average (9). Such negative emotions not only reduce treatment adherence but also accelerate physical functional decline, severely harming the physical and psychological well-being of individuals with multimorbidity (10, 11).

Illness perception is defined as the way an individual perceives a threat to health through cognitive and emotional processing based on personal beliefs and experiences (12). According to Leventhal’s Common-Sense Model (CSM) of self-regulation (13), when individuals develop negative perceptions of their illness—such as believing it has severe consequences, is uncontrollable, or will last a long time—they often experience strong negative emotional responses, including anxiety and depression. This interplay between cognition and emotion directly shapes coping behaviors and psychological adaptation. Conceptually, therefore, negative illness perception serves as an important antecedent of emotional distress, directly triggering or exacerbating negative emotions such as anxiety and depression. According to the CSM, different individuals may form heterogeneous patterns of illness perception based on their unique illness experiences and beliefs, leading to distinct emotional responses and coping strategies (14, 15). Nonetheless, most research has been conducted on individual chronic disorders, and the relationship between illness perception and emotional distress among patients with multimorbidity has not been well studied (16–18). Therefore, identifying heterogeneous subgroups of illness perception among older adults with multimorbidity holds important theoretical value for understanding the mechanisms underlying emotional distress in different patient populations.

Family resilience has become known as a protective factor among the other numerous factors that affect the correspondence between illness perception and emotional distress (19, 20). According to Walsh’s family resilience theory, families with high resilience can alleviate illness-related stress through adaptive mechanisms such as effective communication, shared problem-solving, shared beliefs, and the mobilization of social resources, thereby improving patients’ cognitive and emotional responses to their illness (21–23). However, the mediating effect of family resilience on different illness perception patterns and emotional distress in older adults with multimorbidity has not been well explored. Based on this, we propose that family resilience may mediate the relationship between illness perception and emotional distress. On one hand, when patients hold negative illness perceptions, family communication may decrease or family burden may increase, thereby weakening family resilience. On the other hand, family resilience, as a protective resource, can buffer the negative impact of adverse illness perceptions on emotional distress.

Previous studies have predominantly employed variable-centered approaches to investigate linear relationships between illness perception and emotional distress, often neglecting the potential heterogeneity within patient populations. According to the CSM, illness perception is a multidimensional construct encompassing both cognitive representations and emotional responses to illness. Older adults with multimorbidity, who concurrently manage multiple chronic conditions and experience complex symptoms, treatment burdens, and uncertain disease trajectories, may exhibit markedly different patterns of illness perception. Some patients may maintain relatively positive views of their illness, while others may develop highly pessimistic and less modifiable negative perceptions. This heterogeneity poses challenges for traditional variable-centered methods. In contrast, person-centered latent profile analysis (LPA) can identify latent subgroups characterized by distinct illness perception profiles based on individuals’ response patterns across multiple dimensions (24–27). This approach provides a novel perspective for understanding the intricate structure of illness perception and establishes an empirical foundation for implementing stratified, targeted psychological interventions.

Therefore, the proposed research hypothesized (1) to determine the heterogeneous profiles of illness perception in older adults with multimorbidity via LPA, (2) to compare the patterns of family resilience and emotional distress (anxiety and depression) by profiles of illness perception, and (3) considering the findings of the profile, to consider the indirect effect of family resilience between the categories of illness perception and emotional distress. The results will be considered as a reference in shaping individualized, focused psychosocial intervention strategies for older adults with multimorbidity, depending on their psychological traits.

Based on previous research, we propose the following hypotheses:

H1: LPA will identify distinct latent subtypes of illness perception among older adults with multimorbidity.

H2: Family resilience may serve as a mediating variable in the relationship between illness perception and emotional distress.

## 2 Methods

### 2.1 Participants and recruitments

This study utilized a convenience sampling method to recruit participants from the departments of Geriatrics, Cardiology, Respiratory Medicine, and Endocrinology at three tertiary hospitals in Urumqi, Xinjiang Uygur Autonomous Region, between October 2025 and January 2026. All patients were in a clinically stable condition during data collection, as assessed and confirmed by their attending physicians. The inclusion criteria were as follows: (1) age ≥ 60 years; (2) having two or more chronic conditions, which were determined using the Charlson Comorbidity Index (28), and patients who had two or more conditions were considered to have multimorbidity; and (3) mental alertness and voluntary willingness to participate. The exclusion criteria were as follows: (1) severe cognitive impairment or disturbance of consciousness; (2) being in the acute phase of a disease, such as acute myocardial infarction or acute respiratory failure; and (3) refusal to participate.

A total of 437 questionnaires were sent, and 420 valid questionnaires were received, giving a response rate of 96.1%. Before the survey was administered, the researchers clarified the reasons for and importance of the study. Informed consent was obtained from the participants, who then completed the questionnaire in writing, and those who had problems with either vision or writing were given on-site help.

### 2.2 Sample size

For latent profile analysis (LPA), the required sample size is influenced by several factors, including the number of indicators, the actual number of classes, the distances between classes, and the model selection method. Simulation studies conducted by Tein et al (29). indicate that when the number of indicators ranges from three to five, a sample size exceeding 300 ensures stable and accurate classification. In this study, the eight items of the questionnaire served as indicator variables for LPA, and the sample size of 420 meets this requirement. Furthermore, Tein et al. observed that when the distances between classes are moderate, a sample size between 250 and 500 is adequate for methods such as BIC, LMR, or BLRT to accurately identify the number of classes. Thus, the sample size in this study is sufficient to support stable classification in LPA.

Regarding mediation analysis, scholars such as MacKinnon et al (30). suggest that when employing the bias-corrected bootstrap method to test indirect effects, a sample size of 200 to 400 provides adequate statistical power. In summary, the inclusion of 420 participants in this study ensures sufficient statistical power for the analyses.

### 2.3 Instrument

#### 2.3.1 Demographic characteristics

The questionnaire was divided into two sections: (1) Sociodemographic variables, including age, gender, marital status, educational attainment, monthly household income, and primary caregiver; (2) Clinical characteristics that consisted of the number of comorbid conditions, time since diagnosis, Activities of Daily Living (ADL) score, and Visual Analogue Scale (VAS) pain score.

#### 2.3.2 Measurement of BIPQ

The Brief Illness Perception Questionnaire (BIPQ) was originally developed by Broadbent et al (31). and translated into Chinese by Chen et al (32). The questionnaire was used to assess patients’ cognitive representations and emotional responses to illness. This questionnaire has nine questions which cover three dimensions: cognition (items 1 to 5), emotion (items 6 and 8) and comprehension (item 7). The ninth element was an open-ended question. The respondents rated each question on a 10-point Likert scale. All items were positively scored, the possible score will be between 0 and 80, with higher scores indicating a negative attitude toward the illness. This scale has been widely used among older adults with chronic conditions, with a Cronbach’s α of 0.84, demonstrating good reliability and validity (14). The alpha of Cronbach’s BIPQ in this study was 0.917.

#### 2.3.3 Measurement of resilience

The concept of family resilience, suggested by Walsh (22), is concerned with how families that have a mentally ill member, dysfunctional dynamics, etc., may confront the previous disregard towards family resilience and live meaningful lives. Li et al (33). translated and culturally modified the Chinese version of the Family Resilience Assessment Scale (FRAS-C) into Chinese. The scale has 32 questions (representing three dimensions) that include the following: family communication and problem-solving (FCPS), utilizing social resources (USR), and maintaining a positive outlook (MPO). The items were rated on a 4-point Likert scale (1=Strongly Disagree, 4=Strongly Agree). The overall score ranges from 32 to 128, with a higher score implying a more resilient family with a greater ability to handle stressful situations. It has also been validated in patients with chronic diseases, achieving a Cronbach’s α as high as 0.964, indicating strong reliability and validity (34). In this study, Cronbach’s α for this scale was 0.842.

#### 2.3.4 Measurement of emotional distress

The Hospital Anxiety and Depression Scale (HADS), first presented by Zigmond and Snaith in 1983, is a screening tool which assesses emotional distress in hospital inmates (35). The scale consists of 14 items, which are divided into two different subscales: the Anxiety Subscale (HADS-A) and the Depression Subscale (HADS-D), with each subscale containing 7 items that measure the state of anxiety and depression. Everything is scored using a Likert scale that has 4 points with a range of 0 to 3, which results in a total score for each subscale ranging between 0 and 21. High scores would indicate a high level of emotional distress. The HADS has been widely applied in studies of older adults with chronic conditions and cancer patients (36, 37). Previous studies have shown that this scale is well-validated in older chronic disease populations, with a Cronbach’s α of 0.85 (37). In this study, Cronbach’s alpha of 0.882 was obtained for the scale.

### 2.4 Statistical analysis

First, descriptive statistics were performed, with results presented as means, standard deviations, percentages, and effect sizes (38). Second, tests of normality were conducted, and the data were not normally distributed. Consequently, Spearman’s correlation was used to test the level of linear correlations between the following variables: illness perception, family resilience, and emotional distress. A correlation heatmap was created. Third, LPA was applied to determine possible subgroups with specific patterns of illness perception characteristics. The analysis started with a one-class model and proceeded until the fit indices did not show significant improvement. The fit indexes were the Akaike Information Criterion (AIC), Bayesian Information Criterion (BIC), adjusted BIC (aBIC), Entropy, Lo-Mendell-Rubin Likelihood Ratio Test (LMR-LRT), Bootstrap Likelihood Ratio Test (BLRT), and class probabilities. Smaller values of AIC, BIC, and aBIC and larger entropy (>0.8) indicated a better model fit (39). Univariate and multivariate regression analyses were then conducted to determine the factors related to the profiles obtained based on LPA, whose findings are presented in a forest plot. A Sankey diagram was also created to depict the distribution of profiles based on various characteristics. Bayesian factor analysis was employed to examine differences in HADS scores across the LPA-derived profiles (40). Fourth, Harman’s single-factor test was employed to evaluate the presence of potential common method bias (41). A significant common method bias was considered absent if a single factor explained less than 40% of the total variance. Fifth, differences in FR and HADS among patients with different illness perception categories were compared. Finally, based on the two dimensions of HADS (anxiety and depression), the PROCESS macro (Model 4) was utilized to examine the mediating effect of FR between BIPQ based on LPA and anxiety and depression respectively (42). To adequately control for the potential influence of demographic variables on the mediation model, demographic characteristics that showed significant correlations with illness perception categories and HADS scores after testing were included as covariates in the final analysis. The significance level was set at α = 0.05. Data analysis in this study was performed using SPSS 27.0, Mplus 8.3, JASP 0.19.3, and R 4.5.2.

## 3 Results

### 3.1 Demographic and clinical characteristics

Among the 420 participants, the majority were male (52.14%) and aged ≥ 65 years (76.67%). Univariate analysis revealed significant differences in emotional distress across education levels (H = 28.302, *P* < 0.001, ϵ² = 0.068), with the highest scores in the college or above group (12.93 ± 4.47). Monthly household income was also significantly associated with emotional distress (H = 23.561, *P* < 0.001, ϵ² = 0.056), with the middle−income group reporting the highest distress (12.63 ± 4.52). In addition, significant differences were observed for age (Z = 2.541, *P* = 0.011, r = 0.124), time since diagnosis (Z = 2.891, *P* = 0.004, r = 0.141), ADL score (Z = 2.503, *P* = 0.012, r = 0.122), and VAS pain score (Z = 3.760, *P* < 0.001, r = 0.184). Gender also showed a significant difference (Z = –2.741, *P* = 0.006, r = 0.134), with females reporting higher emotional distress than males. No significant differences were found for marital status, primary caregiver, or number of chronic diseases (*P* > 0.05). Detailed demographic and clinical characteristics are presented in **Table 1**.

**Table 1:** Demographics, clinical characteristics, and emotional distress levels (*N* = 420).

Variables	Classification	n (%)	Emotional distress (M ± SD)	Test statistic	P	Effect size
Gender	Female	201 (47.86)	11.97 ± 4.47	Z = -2.741	0.006	r = 0.134
	Male	219 (52.14)	10.75 ± 4.62			
Education level	Primary or below	173 (41.19)	11.10 ± 4.56	H = 28.302	< 0.001	ϵ² = 0.068
	Secondary	116 (27.62)	9.88 ± 4.20			
	College or above	131 (31.19)	12.93 ± 4.47			
Marital status	Married	118 (28.10)	11.36 ± 4.57	Z = 0.134	0.893	r = 0.007
	Other	302 (71.90)	11.32 ± 4.59			
Monthly household income	< 2000	145 (34.52)	11.11 ± 4.37	H = 23.561	< 0.001	ϵ² = 0.056
	2000–4000	154 (36.67)	12.63 ± 4.52			
	> 4000	121 (28.81)	9.95 ± 4.48			
Primary caregiver	Spouse	47 (11.19)	12.74 ± 4.82	H = 5.660	0.059	ϵ² = 0.014
	Family	111 (26.43)	11.42 ± 4.49			
	Other	262 (62.38)	11.04 ± 4.54			
Age (years)	< 65	98 (23.33)	10.34 ± 4.22	Z = 2.541	0.011	r = 0.124
	≥ 65	322 (76.67)	11.64 ± 4.65			
Number of chronic diseases	Two	234 (55.71)	11.06 ± 4.73	Z = 1.272	0.203	r = 0.062
	Two or more	186 (44.29)	11.68 ± 4.37			
Time since diagnosis	< 1 year	74 (17.62)	9.91 ± 4.59	Z = 2.891	0.004	r = 0.141
	≥ 1 year	346 (82.38)	11.64 ± 4.53			
VAS pain score	< 4	202 (48.10)	10.45 ± 4.70	Z = 3.760	< 0.001	r = 0.184
	≥ 4	218 (51.90)	12.15 ± 4.32			
ADL score	≥ 60	405 (96.43)	11.22 ± 4.56	Z = 2.503	0.012	r = 0.122
	< 60	15 (3.57)	14.27 ± 4.18			

### 3.2 Correlations among illness perception, family resilience, and emotional distress

**Figure 1:**
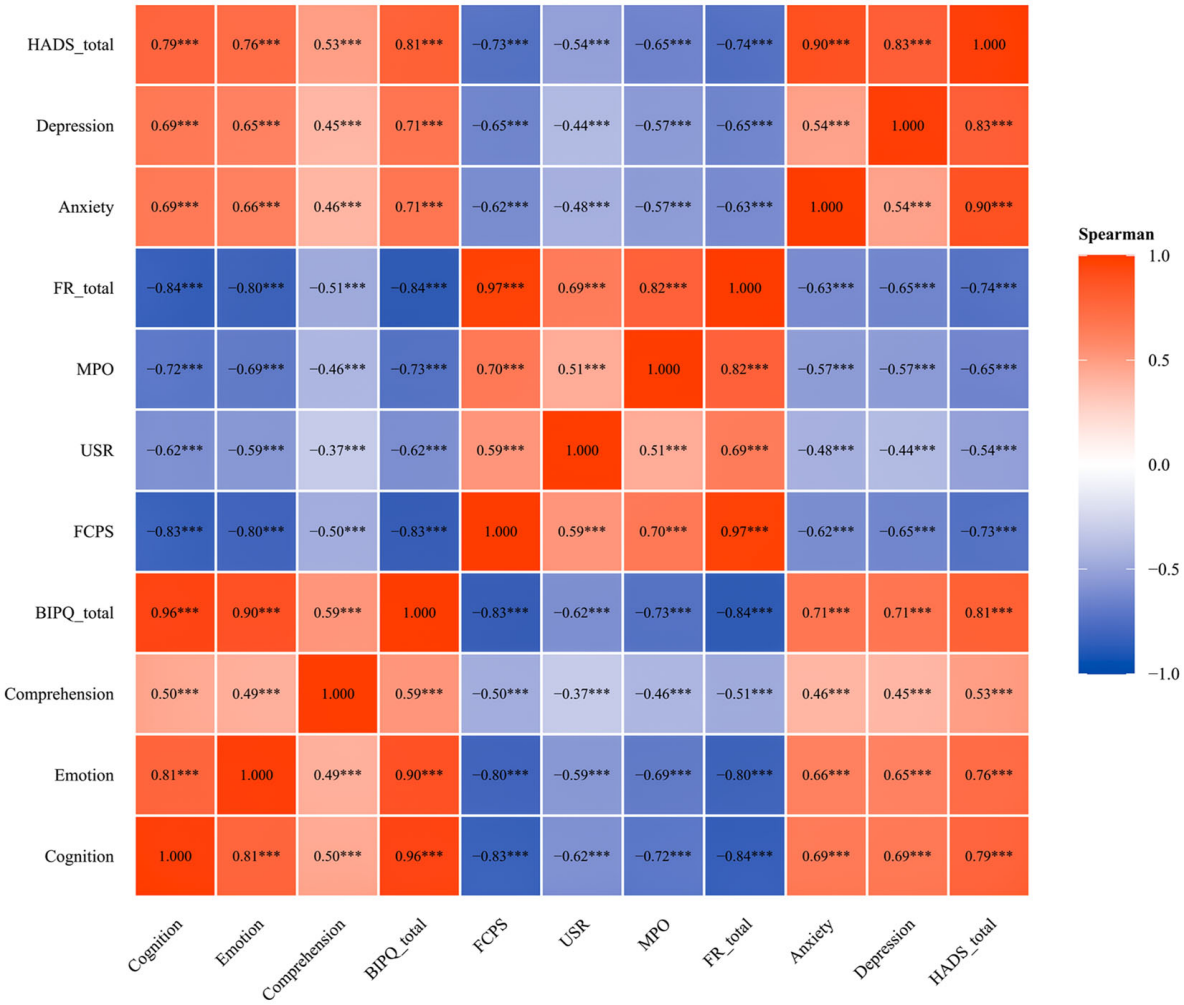
Heatmap of Spearman correlations among illness perception, family resilience, and emotional distress. *** denotes p<0.001.

Harman’s single-factor analysis was adopted to test the potential existence of common method bias. The findings showed that the first factor accounted for 27.52% of the total variance, indicating that the impact of common method bias was negligible in this research. As shown by the results of the normality tests, the scores associated with illness perception, emotional distress, and family resilience showed high skewness (*P* < 0.05), thus the application of the Spearman correlation analysis. The findings showed that: (1) illness perception had a significant positive relationship with emotional distress (r = 0.81, *P* < 0.001); (2) there was a significant negative relationship between family resilience and emotional distress (r = -0.74, *P* < 0.001); and (3) there was a significant negative relationship between illness perception and family resilience (r = -0.84, *P* < 0.001). The results of the Spearman correlation analysis are represented in a heatmap as in **Figure 1**. Additionally, diagnostics for multicollinearity were performed, revealing a VIF of 5.86, which does not indicate any severe multicollinearity concerns.

### 3.3 LPA of illness perception

**Figure 2:**
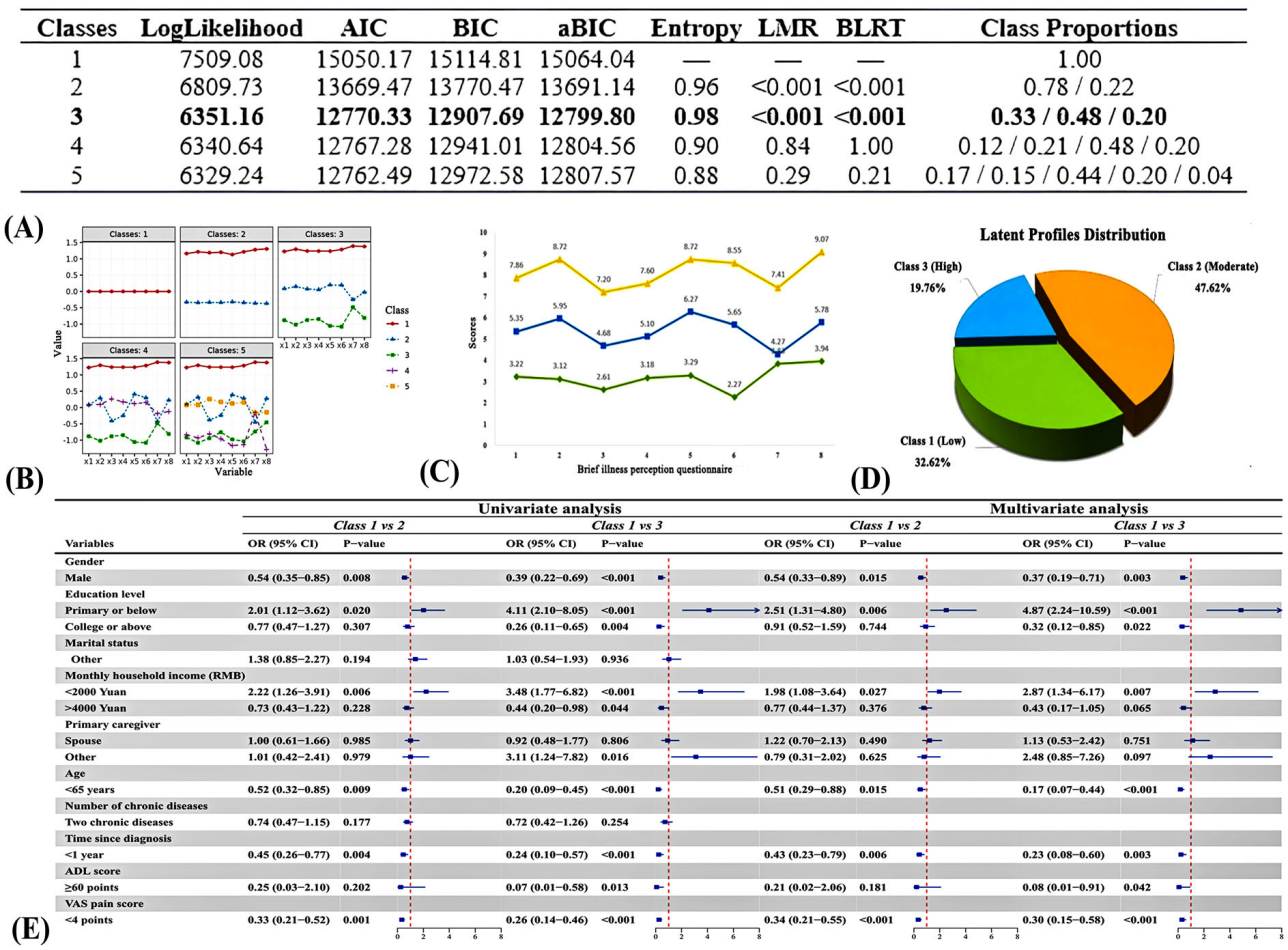
**(A)** Fit indices for the latent profile models. **(B)** Curves illustrating model fit. **(C)** The three final latent classes were identified. **(D)** Distribution of the three latent classes. **(E)** Univariate and multivariate logistic regression analyses based on LPA.

**Figure 3:**
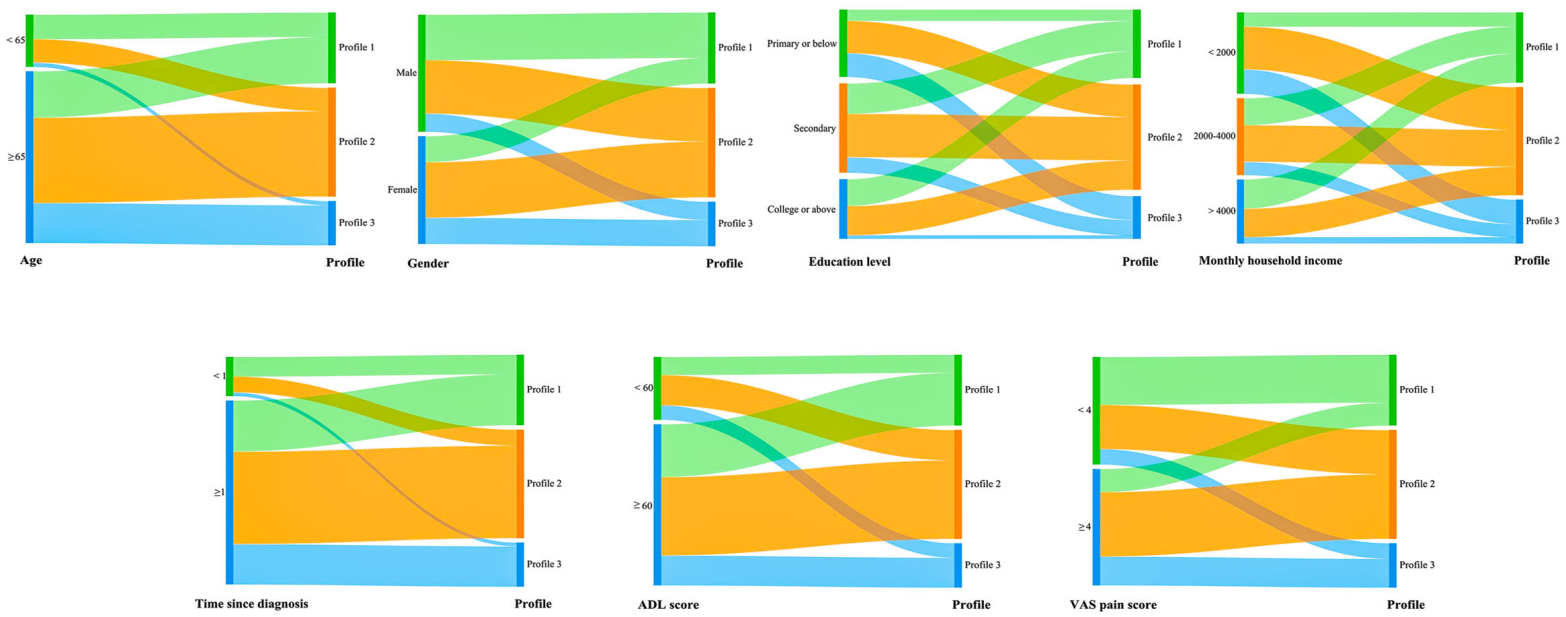
The Sankey diagram visually presents both the distribution of characteristics and their association with outcomes through data flow: the left-hand nodes represent the input characteristic categories (the significant independent variables included in this study), and the right-hand nodes represent the output categories (the three latent profiles). The bands depict data flows, the width of which corresponds to the strength of the flow. The left-to-right direction intuitively reveals how different characteristic variables converge into each outcome profile.

As the number of profiles increased, the values of AIC, BIC, and aBIC decreased. The three-profile model was identified as optimal due to its lowest AIC, BIC, and aBIC values, highest entropy (approaching 1), and statistically significant results from the LMR and BLRT tests (both *p* < 0.001). Detailed fit indices are presented in **Figure 2A**. A total of five profile solutions were estimated, as illustrated in **Figure 2B**. The three illness perception profiles derived from LPA and their respective proportions are displayed in **Figures 2C, D**. Item scores across the three subgroups demonstrated a gradient pattern from low to high, with all items showing ordered increases. Accordingly, the three profiles were labeled as “low illness perception” (32.62%, Class 1), “moderate illness perception” (47.62%, Class 2), and “high illness perception” (19.76%, Class 3). These findings indicate that subgroup differences primarily reflect variations in the severity of illness perception. In other words, the heterogeneity of illness perception in this study is characterized by quantitative rather than qualitative differences. Univariate and multivariate analyses indicated that gender, education level, monthly household income, age, time since diagnosis, Activities of Daily Living (ADL) score, and Visual Analog Scale (VAS) pain score were significant factors distinguishing Class 1 from Class 2, as well as Class 1 from Class 3. These results are detailed in **Figure 2E**, and the coding scheme for the independent variables is detailed in **Table 2**. Further visualization, as shown in **Figure 3**, more clearly illustrates the influence patterns of these factors across distinct latent subgroups.

**Table 2:** Variable assignment.

Variables	Assignment
Age	1 = <65, 2 =≥65
Gender	1 = Female, 2 = Male
Education level	1 = Primary or below, 2 = Secondary, 3 = College or above
Marital status	1 = Married, 2 = Other
Primary caregiver	1 = Spouse, 2 = Family, 3 = Other
Monthly household income	1 = <2000, 2 = 2000-4000, 3 = >4000
Number of chronic diseases	1 = Two chronic diseases, 2 = Two or more chronic diseases
Time since diagnosis	1 =<1 year, 2 = ≥1 year
ADL score	1 = < 60, 2 = ≥ 60
VAS pain score	1 = <4, 2 = ≥4

### 3.4 Differences in HADS scores across LPA-derived profiles

**Figure 4:**
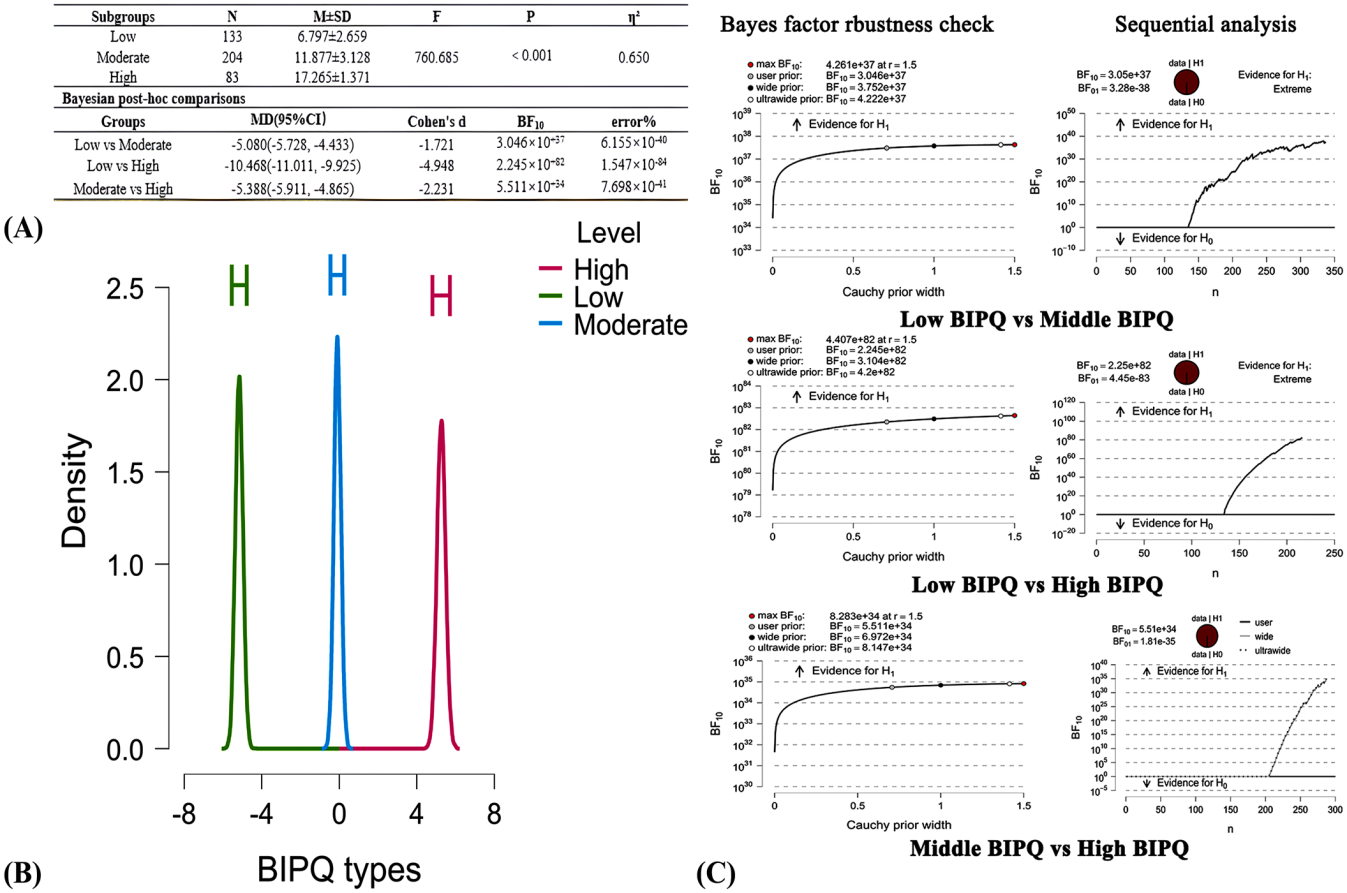
**(A)** Bayesian factor analysis of HADS score differences across LPA profiles. **(B)** Posterior distribution of ANOVA means. **(C)** Bayesian factor analysis inference plot.

Due to the non-normal distribution of the data and the violation of homogeneity of variance, Welch’s ANOVA was employed for group comparisons. The results revealed statistically significant differences in HADS scores across groups (F = 760.685, *P* < 0.001). Specifically, significant differences were observed between the low and moderate BIPQ groups (BF_10_ = 3.046 × 10^+37^, d = −1.721), between the low and high BIPQ groups (BF_10_ = 2.245 × 10^+82^, d = −4.948), and between the moderate and high BIPQ groups (BF_10_ = 5.511 × 10^+34^, d = −2.231). Details are presented in **Figure 4**.

### 3.5 Differences in FR and HADS scores across LPA-derived profiles

**Figure 5:**
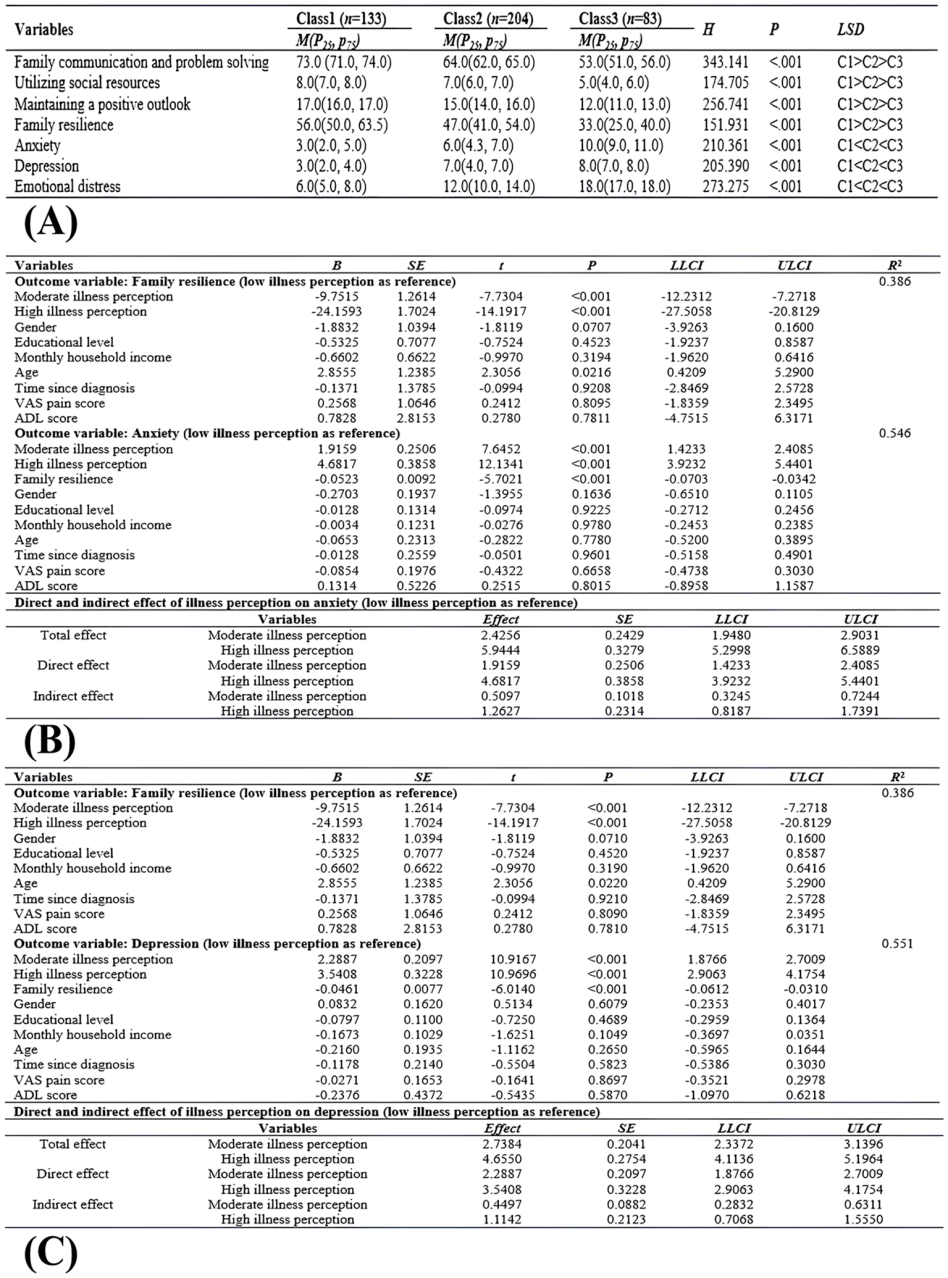
**(A)** Differences in family resilience and emotional distress scores across illness perception profiles. **(B)** The mediating role of family resilience in anxiety. **(C)** The mediating role of family resilience in depression.

As shown in **Figure 5A**, significant differences in scores of family resilience and emotional distress were observed among the three groups (*P* < 0.001). *Post hoc* pairwise comparisons revealed that scores across all dimensions of family resilience gradually decreased from the low to the high illness perception group (Class 1 > Class 2 > Class 3, *P* < 0.001). In contrast, scores on the two dimensions of emotional distress gradually increased from the low to the high illness perception group (Class 1 < Class 2 < Class 3, *P* < 0.001).

### 3.6 Mediating effect of FR based on latent profile analysis

**Figure 6:**
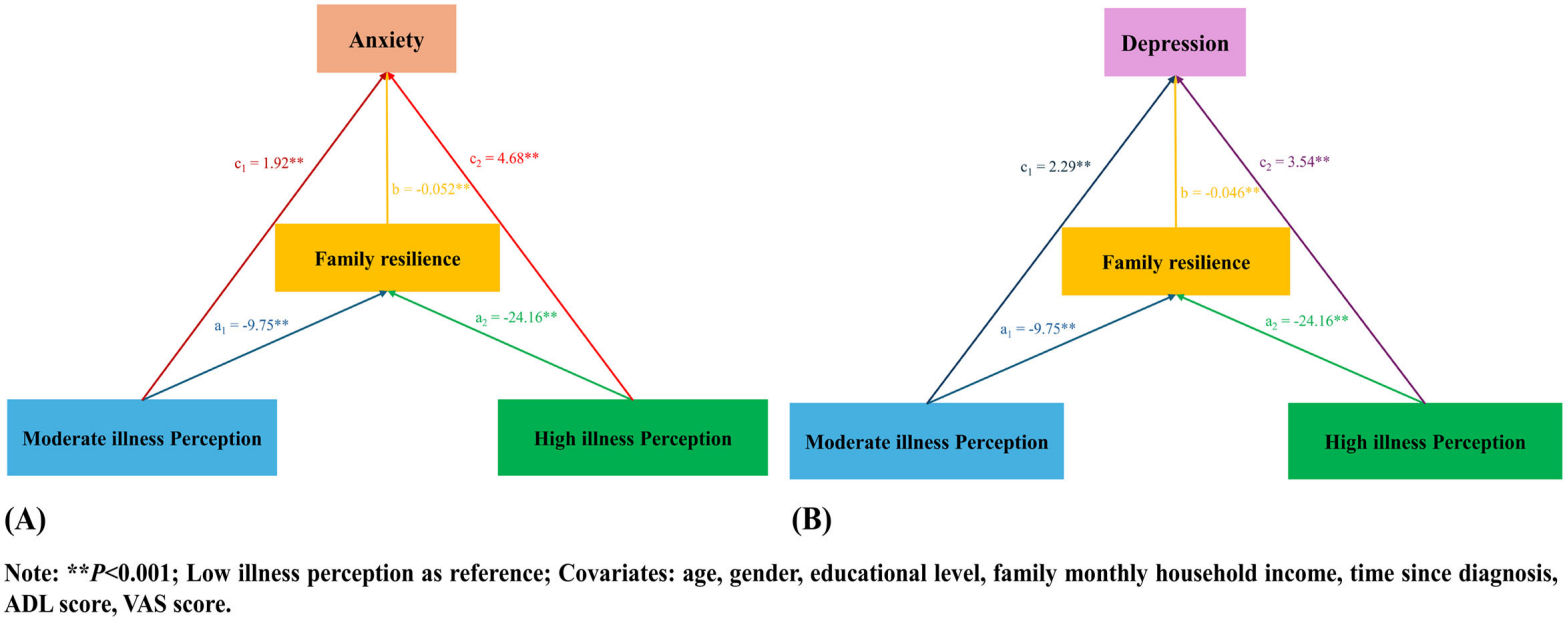
**(A)** Mediating effects of family resilience on anxiety. **(B)** Mediating effects of family resilience on depression.

Separate mediation models were constructed with the low illness perception group as the reference category, family resilience as the mediator, and the anxiety and depression subdimensions of emotional distress as the outcome variables. Additionally, considering the sociodemographic factors and clinical characteristics that influence emotional distress scores, we included gender, education level, monthly household income, age, time since diagnosis, ADL score, and VAS pain score as covariates in the analysis. The results showed that: (1) Anxiety dimension (95% bootstrap confidence intervals): Moderate disease perception group: total effect (1.9480, 2.9031), direct effect (1.4233, 2.4085), indirect effect (0.3245, 0.7244); High disease perception group: total effect (5.2998, 6.5889), direct effect (3.9232, 5.4401), indirect effect (0.8187, 1.7391). (2) Depression dimension (95% bootstrap confidence intervals): Moderate disease perception group: total effect (2.3372, 3.1396), direct effect (1.8766, 2.7009), indirect effect (0.2832, 0.6311); High disease perception group: total effect (4.1136, 5.1964), direct effect (2.9036, 4.1754), indirect effect (0.7068, 1.5550). This results indicated that family resilience served as a significant partial mediator between illness perception and emotional distress. The specific findings are represented in **Figures 5B, C**, and a diagram of the mediation pathways is represented in **Figure 6**.

## 4 Discussion

This study identified heterogeneity in illness perception among older adults with multimorbidity, revealing three distinct subtypes: low, moderate, and high illness perception. The findings further confirm the partial mediating role of family resilience in the relationship between illness perception and emotional distress, specifically anxiety and depression. These results underscore the importance of shifting the clinical focus from the individual to the family system and integrating the enhancement of family resilience into psychosocial support interventions.

First, we identified three latent classes of illness perception among older patients with multimorbidity: low (32.6%), moderate (47.6%), and high (19.8%). This distribution suggests that a considerable proportion of older adults with multimorbidity exhibit relatively negative cognitive and emotional responses to their health status. Illness perception represents a subjective construction of health threats, shaped largely by personal experiences, beliefs, and encounters with illness (16). Multimorbidity entails the management of more complex symptoms, more frequent healthcare visits, and a higher overall disease burden. These challenges may heighten patients’ negative expectations regarding the controllability, consequences, and progression of their illnesses, thereby deepening their concerns about disease outcomes. In addition, disease-related symptoms, treatment burden, and the associated financial stress may severely disrupt daily life and further exacerbate negative emotions (43, 44). In this study, we found a positive correlation between illness perception and emotional distress: patients with multimorbidity tend to develop negative illness perceptions, and such persistent negative cognitions contribute to significant emotional distress, leading to feelings of worry, fear, pessimism, and even hopelessness. Therefore, helping patients develop an objective and accurate illness perception plays a key role in alleviating emotional distress, improving disease prognosis, and enhancing quality of life. By using latent profile analysis, this study transcends the linear relationships typically explored in traditional variable-centered research. It facilitates a more nuanced identification of latent patient subgroups and establishes an empirical foundation for the implementation of stratified, targeted psychological interventions (16, 43, 44).

Demographic and disease-related characteristics significantly influenced illness perception and emotional distress. Female patients reported notably higher levels of emotional distress, which may be attributed to their heightened sensitivity to health issues and a greater propensity to express emotional distress within sociocultural contexts (45). Additionally, women often face multiple stressors associated with family caregiving roles (46, 47). In terms of education level, older patients with higher educational attainment exhibited better comprehension of health information. However, they also recorded higher emotional distress scores. This finding suggests that, within the framework of clinical multimorbidity management, patients with higher education may experience increased anxiety and pessimism due to their more profound understanding of their diseases (48). Economically, lower family income emerged as a significant risk factor for emotional distress, aligning with prior literature (49, 50). High treatment costs and extended recovery periods can impose substantial financial burdens, directly exacerbating worries and feelings of helplessness regarding disease prognosis among patients and their families (51, 52). In contrast, hospitalized older patients under the age of 65 displayed lower emotional distress and a more positive illness perception, likely attributable to their better physiological reserves and enhanced social adaptability (53–55). Regarding disease characteristics, patients with a diagnosis duration exceeding one year exhibited more severe negative illness perceptions, potentially resulting from the financial pressures and disruptions to normal life associated with long-term treatment (56–59). Lower pain levels and improved functioning in activities of daily living were associated with a more positive illness perception and reduced emotional distress (60, 61). This finding underscores the protective role of symptom management and functional maintenance in promoting mental health. The interpretations outlined above concerning the associations between demographic and disease-related factors, illness perception, and emotional distress are primarily derived from prior studies and theoretical reasoning, rather than being directly tested for causality in the current investigation. Future research utilizing longitudinal or interventional designs is necessary to further validate the causal relationships among these variables and to examine the underlying mechanisms (62–66).

Based on the above findings, this study offers the following practical implications for clinical psychological care of older adults with multimorbidity: For highly educated patients, it is recommended that clinical guidance emphasize the controllability of the disease to help alleviate negative emotions during treatment (67). For low-income populations, it is suggested that the government increase investment in medical insurance, expand coverage, and raise reimbursement rates to reduce their financial burden (49, 51). Enhanced emotional support and functional rehabilitation should be prioritized for female and older patients (45–47). Attention should also be given to managing somatic symptoms such as pain while maintaining activities of daily living (61, 62). Patients and their families should be actively involved in treatment planning, and efforts should be made to strengthen family empowerment and skills training to enhance caregiving capacity and improve communication regarding the illness. Furthermore, given the multi-ethnic cultural context of Xinjiang, it is suggested that a hospital-community-family network be established to foster interdisciplinary collaboration, shifting the intervention model from a disease-centered approach to a holistic care model that addresses physical, psychological, social, and spiritual health. Future research, such as policy intervention trials or community-based controlled studies, is needed to further evaluate the feasibility and effectiveness of these recommendations.

Second, the study revealed that family resilience serves as a significant partial mediator between illness perception and emotional distress, thereby supporting hypothesis 2. Specifically, in patients exhibiting moderate to high levels of illness perception, negative illness cognition markedly diminished the adaptive capacity of the family system, or family resilience, which subsequently resulted in heightened anxiety and depressive symptoms (68). This finding is consistent with Walsh’s family resilience theory, which posits that families function as systems capable of mitigating the adverse effects of stressors on their members through adaptive mechanisms such as positive communication, shared belief systems, collaborative problem-solving, and the mobilization of social resources (22). In this study, scores reflecting various dimensions of family resilience exhibited a stepwise decline from low to high illness perception groups, while emotional distress scores demonstrated an opposing trend. This pattern visually underscores the inverse relationship between protective family resources and patient mental health (7, 69). Consequently, clinical interventions should not only focus on altering individual patient cognition but also seek to enhance the family’s overall adaptive capacity in the face of illness-related challenges. This could be achieved, for instance, through family meetings aimed at improving communication or by cultivating skills for collaborative health management (21).

This study employed both frequentist statistics and Bayesian factor analysis when comparing emotional distress across different illness perception subgroups. Bayesian analysis was introduced to provide complementary quantification of evidence to the frequentist results (70). Unlike traditional statistical analysis that only reports p-values, the Bayes factor (BF_10_) quantifies the strength of evidence in favor of the alternative hypothesis (i.e., differences exist between groups) relative to the null hypothesis (i.e., no differences), and is not strictly constrained by the assumption of normal distribution. According to recommendations by Quintana and Williams (40), a BF_10_ greater than 10 indicates strong evidence, and greater than 100 indicates extremely strong evidence. In this study, the BF_10_ for the comparison between the low-perception group and the high-perception group was 2.245 × 10^−8^², far exceeding 100, suggesting extremely strong evidence in support of differences between the two groups. It is noteworthy that Bayesian factor analysis indicated significant differences in emotional distress scores across the different illness perception subgroups (40, 70, 71). This may be partly attributable to the specific characteristics of our study sample, namely older inpatients with multimorbidity from a provincial tertiary hospital in Xinjiang. These individuals generally bear a high disease burden and share similar medical and cultural backgrounds. This homogeneity may lead to limited within-group variation while amplifying between-group differences. Future research should validate these findings in broader, more heterogeneous community-dwelling older populations (45).

In summary, this study elucidates the heterogeneous patterns of illness perception among hospitalized older adults with multimorbidity and identifies a significant mediating pathway through which family resilience affects emotional distress. These findings suggest new directions for clinical practice: when assessing and managing the psychological well-being of older adults with multimorbidity, it is crucial to concurrently evaluate their patterns of illness perception and family functioning (6, 21). For patients demonstrating moderate to high levels of illness perception, intervention strategies should incorporate measures designed to enhance family resilience, such as family conferences and training in family support skills (57). By strengthening the family system, we can more comprehensively and sustainably improve patients’ emotional health and quality of life (47, 72).

## 5 Limitations

Our sample comprised older inpatients with chronic multimorbidity from Xinjiang. Due to similarities in healthcare environments, substantial disease burdens, and shared age and cultural characteristics, this population demonstrated a high degree of homogeneity in their psychological and clinical responses. Notably, within the subgroup exhibiting a heightened illness perception, emotional distress scores were elevated with minimal internal variation. These findings necessitate validation in larger-scale, more diverse community-dwelling older populations. Furthermore, future studies should incorporate qualitative interviews to elucidate nuanced differences in the psychological status of older individuals living with long-term multimorbidity. Additionally, the current study utilized a cross-sectional design. Although mediation analysis was employed to explore pathways, it cannot establish causal inferences regarding the relationships among illness perception, family resilience, and emotional distress. Future longitudinal or interventional studies are required to further clarify these relationships. This research was conducted exclusively in Xinjiang; thus, factors such as ethnic culture, accessibility of medical resources, and family structures may limit the generalizability of the results to other populations and regions. Further multi-center studies across broader geographical areas are essential to enhance generalizability. Although common method bias was assessed, all key variables were obtained through self-reported questionnaires, which may still introduce bias. To strengthen future research, it is recommended to incorporate appropriate physiological indicators as supplementary measures.

## 6 Conclusion

This study identified substantial heterogeneity in illness perception among hospitalized older adults with multimorbidity, categorizing them into three latent subgroups: low, medium, and high. Family resilience exhibited a significant partial mediating effect in the relationship between illness perception and emotional distress, which includes anxiety and depression, thereby indicating an indirect association among these three variables. These findings offer preliminary evidence for the development of individualized psychological interventions tailored to older patients with varying illness perception characteristics. It is important to note that, due to the cross-sectional design of this study, causal relationships among the variables cannot be established. Future research should consider employing longitudinal or interventional study designs to validate these causal pathways and expand the sample size to enhance the external validity of the findings.

## Data Availability

The raw data supporting the conclusions of this article will be made available by the authors, without undue reservation.
